# Efficacy of rezafungin in a case of *Candida* spondylodiskitis

**DOI:** 10.5194/jbji-9-213-2024

**Published:** 2024-10-07

**Authors:** Marin Lahouati, Claire Tinévez, Frédéric Gabriel, Fabien Xuereb, Maxime Lefranc, Frédéric-Antoine Dauchy

**Affiliations:** 1 Service de Pharmacie Clinique, CHU de Bordeaux, 33076 Bordeaux, France; 2 INSERM, Biologie des Maladies Cardiovasculaires, Université de Bordeaux, U1034, 33600 Pessac, France; 3 Centre de Référence des Infections Ostéo-Articulaires complexes (CRIOAc GSO), CHU de Bordeaux, 33076 Bordeaux, France; 4 Service des Maladies Infectieuses et Tropicales, CHU de Bordeaux, 33076 Bordeaux, France; 5 Service de Parasitologie-Mycologie, CHU de Bordeaux, 33076 Bordeaux, France

## Abstract

Rezafungin, which only requires weekly administration, is a potential candidate for difficult-to-treat infections that require long-term antimicrobial treatment, such as bone and joint infections. We report the first case of *Candida glabrata* spondylodiskitis successfully treated with 3 weeks of caspofungin followed by 10 weeks of rezafungin.

## 1 Introduction

Bone and joint infections (BJIs) due to fungal agents are infrequent and challenging to treat, requiring extended antifungal treatment (Gamaletsou et al., 2022). Rezafungin, a next-generation echinocandin derived from anidulafungin, has a similar spectrum to other echinocandins with respect to *Candida* spp. and *Aspergillus* spp. STRIVE and ReSTORE trials led to Food and Drug Administration (FDA) approval of rezafungin for the treatment of candidaemia and invasive candidiasis in adult patients who have limited or no alternative treatment options (Thompson et al., 2024). Unfortunately, to date, there are no data on the efficacy of rezafungin with respect to BJIs, as these infections were excluded from the phase-III clinical trials. However, rezafungin has an innovative pharmacokinetic property, with enhanced tissue penetration and an extended elimination half-life of 130 h (Sandison et al., 2017). This long elimination half-life allows for a weekly intravenous infusion administration mode. This combination of factors makes rezafungin a potential candidate for difficult-to-treat infections that require long-term antimicrobial treatment, such as BJIs or endocarditis, in the same way that dalbavancin or oritavancin is used for treating *Staphylococcus* spp. infections. Here, we present the case study of a severe fungal BJI successfully treated with rezafungin.

## 2 Case study

**Figure 1:**
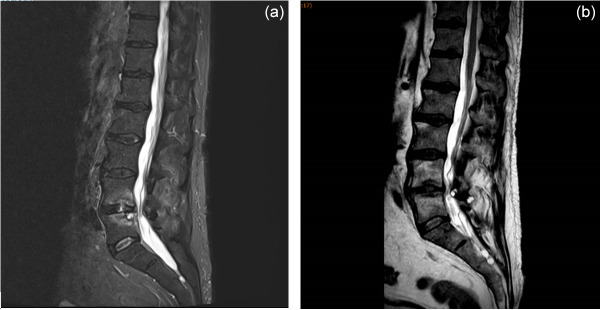
MRI sagittal planes of L4–L5 spondylodiskitis showing **(a)** bone erosions and epiduritis at diagnosis and **(b)** the resolution of lesions 5 months after the end of rezafungin treatment.

A 63-year-old man with a history of diabetes, hypertension, and benign prostatic hypertrophy had a renal lithiasis, which led to obstructive pyelonephritis treated with the placement of a double-J stent in the ureter and antibiotherapy. A total of 1 month later, the double-J stent was removed. A few days later, the patient began to experience fever associated with back pain. A spine magnetic resonance imaging (MRI) scan revealed L4–L5 spondylodiskitis (Fig. 1).

The patient was hospitalized in the infectious diseases unit of Bordeaux University Hospital. Upon admission, the patient had no fever, severe lower-back pain, a C-reactive protein (CRP) value of 8.1 mg L^-1^, a leucocyte value of $7.2\times 10^{9}$ L^-1^, and a slightly positive (7.6 pg mL^-1^ for a threshold of 7 pg mL^-1^) serum β -D-glucan (BDG) level (FUJIFILM Wako Chemicals Europe, Germany). Multiple L4–L5 radio-guided bone biopsies were performed, and all five biopsies yielded *Candida glabrata* (*Nakaseomyces glabratus*) in culture. Specific bacterial and mycobacterial cultures remained sterile. The *C. glabrata* strain exhibited cross-resistance to azole in vitro with a minimum inhibitory concentration (MIC) for fluconazole and voriconazole at 256 and 8 mg L^-1^, respectively. The MIC for caspofungin was 0.064 mg L^-1^, whereas it was 0.016 mg L^-1^ for micafungin; therefore, the isolate was presumed to be susceptible to rezafungin as well (Pfaller et al., 2016).

An intravenous treatment with caspofungin was carried out (70 mg d^-1^) for 3 weeks and was then followed by rezafungin (a first dose of 400 mg followed by a 200 mg dose weekly for 10 weeks). Rezafungin was well tolerated for the entire treatment period. Serum BDG was negative ( <3 pg mL^-1^) after 2 weeks of caspofungin treatment. Due to clinical improvement and negative serum β -D-glucan results, we did not prolong antifungal treatment beyond 3 months. MRI examinations (performed 2 and 5 months after the conclusion of antifungal treatment) showed the regression of lesions (Fig. 1). At 5 months following the conclusion of antifungal treatment, the patient had gained weight and back pain had disappeared. The outcome was still favorable 3 months later (at the 8-month follow-up), and the patient will be monitored for a total duration of 2 years.

## 3 Discussion

Cases of *Candida* spp. osteomyelitis are infrequent; thus, there are no randomized clinical trials to compare treatments for these BJIs. Gamaletsou et al. (2012) reported 207 cases of *Candida* spp. osteomyelitis, 51 % of which were located on vertebras. *C. glabrata* was only implicated in 17 cases (8 % of instances). The median treatment duration was 90 d, and treatment primarily involved an antifungal combination (45 % of instances), including amphotericin B or azoles. Azoles are often the only oral antifungal treatment available. However, approximately 30 % of the *C. glabrata* strains isolated in France are resistant to fluconazole (Desnos-Ollivier et al., 2021). In addition, azoles are known to have drug interactions that may limit their use, especially in frail, elderly patients receiving polypharmacotherapy. Thus, alternative options are poor. Amphotericin B may be an alternative, but there is a high risk of adverse events, such as nephrotoxicity, leading to early treatment discontinuation and promoting relapse. Caspofungin, micafungin, or anidulafungin may also be considered as alternatives (Gamaletsou et al., 2022); however, daily intravenous administration can lead to catheter-associated complications and decrease patients' quality of life. In these difficult-to-treat infections, rezafungin seems to be a promising therapeutic option. The higher and longer exposures achieved with rezafungin in vivo should have important benefits with respect to the prevention of biofilm-associated nosocomial infections (e.g., catheter-related infections) and reduce the length of hospital stays. Viceconte et al. (2024) reported a case in which *C. parapsilosis* spondylodiskitis was successfully treated by 26 weeks of rezafungin. It is of note that rezafungin was used after 10 weeks of effective antifungal treatment (anidulafungin, voriconazole, and liposomal amphotericin B), making the intrinsic effectiveness of rezafungin difficult to extrapolate.

Here, we report case in which *C. glabrata* spondylodiskitis was successfully treated by 3 weeks of caspofungin followed by 10 weeks of rezafungin. Larger series of case studies and larger cohorts are required to evaluate the real-life efficacy of rezafungin.

## Data Availability

The data set is available from the corresponding author upon reasonable request.

## References

[bib1.bib1] Desnos-Ollivier M, Lortholary O, Bretagne S, Dromer F (2021). Azole Susceptibility Profiles of More than 9,000 Clinical Yeast Isolates Belonging to 40 Common and Rare Species. Antimicrob Agents Chemother.

[bib1.bib2] Gamaletsou MN, Kontoyiannis DP, Sipsas NV, Moriyama B, Alexander E, Roilides E, Brause B, Walsh TJ (2012). Candida Osteomyelitis: Analysis of 207 Pediatric and Adult Cases (1970–2011). Clin Infect Dis.

[bib1.bib3] Gamaletsou MN, Rammaert B, Brause B, Bueno MA, Dadwal SS, Henry MW, Katragkou A, Kontoyiannis DP, McCarthy MW, Miller AO, Moriyama B, Pana ZD, Petraitiene R, Petraitis V, Roilides E, Sarkis J-P, Simitsopoulou M, Sipsas NV, Taj-Aldeen SJ, Zeller V, Lortholary O, Walsh TJ (2022). Osteoarticular Mycoses. Clin Microbiol Rev.

[bib1.bib4] Pfaller MA, Messer SA, Rhomberg PR, Jones RN, Castanheira M (2016). Activity of a long-acting echinocandin, CD101, determined using CLSI and EUCAST reference methods, against *Candida* and *Aspergillus* spp., including echinocandin- and azole-resistant isolates. J Antimicrob Chemother.

[bib1.bib5] Sandison T, Ong V, Lee J, Thye D (2017). Safety and Pharmacokinetics of CD101 IV, a Novel Echinocandin, in Healthy Adults. Antimicrob Agents Chemother.

[bib1.bib6] Thompson GR, Soriano A, Honore PM, Bassetti M, Cornely OA, Kollef M, Kullberg BJ, Pullman J, Hites M, Fortún J, Horcajada JP, Kotanidou A, Das AF, Sandison T, Aram JA, Vazquez JA, Pappas PG (2024). Efficacy and safety of rezafungin and caspofungin in candidaemia and invasive candidiasis: pooled data from two prospective randomised controlled trials. Lancet Infect Dis.

[bib1.bib7] Viceconte G, Buonomo AR, Esposito N, Cattaneo L, Somma T, Scirocco MM, Mainolfi CG, Gentile I (2024). Salvage Therapy with Rezafungin for Candida parapsilosis Spondylodiscitis: A Case Report from Expanded Access Program. Microorganisms.

